# Prevalence and Risk Factors of Self-Reported Perfume Sensitivity in Saudi Arabia

**DOI:** 10.3390/healthcare9101248

**Published:** 2021-09-22

**Authors:** Meshael Alrasheed, Omar Albalawi, Mohammed Aljallal, Amani S. Alqahtani

**Affiliations:** Saudi Food and Drug Authority, Riyadh 13513, Saudi Arabia; ombalawi@sfda.gov.sa (O.A.); majallal@sfda.gov.sa (M.A.); as.qahtani@sfda.gov.sa (A.S.A.)

**Keywords:** perfumes, fragrances, sensitivity, respiratory, dermatology, asthma

## Abstract

Perfumes are widely used products; however, several fragrance substances used in perfuming are well-established allergenic substances and have been attributed to various adverse health reactions. The nature and significance of perfume sensitivity reactions have not been thoroughly investigated. Therefore, this study aimed to identify the prevalence, nature, and associated risk factors of self-reported perfume sensitivity among the general population in Saudi Arabia. A nationally representative cross-sectional study was conducted among adults in Saudi Arabia in October 2020. Significant associated risk factors were explored using multivariate regression analyses. A total of 1078 participants completed the survey, with a mean age of 36.7 years (SD ± 10.36). Perfume sensitivity reactions were reported in 14.6% of participants. From among these participants, 17.8% reported moderate to severe reactions. Respiratory and skin symptoms were the most reported reactions, with total rates of 40.1% and 35.7%, respectively. History of asthma (OR = 3.2, 95%CI 1.88–4.37, *p* < 0.001) and the use of counterfeit perfume products (OR = 1.9, 95%CI 1.23–2.94, *p* < 0.003) were significantly associated with a higher risk of perfume sensitivity. Our study revealed that a considerable number of the general population in Saudi Arabia has experienced adverse health reactions due to perfume products. The enormous volume of the perfume market thus necessitates further quantitative analysis studies to determine the presence of allergenic fragrance substances in perfumes.

## 1. Introduction

Fragrance sensitivity is a health condition characterized by the experience of adverse health effects from exposure to a fragranced product [1]. Most commonly reported fragrance reactions include skin, respiratory, neurological, and nasal symptoms [2]. Perfumes have been reported as one of the fragranced products that contribute to fragrance sensitivity in several international studies [3,4]. A total rate of 32.2% of the public reported sensitivity to fragranced products, including perfumes, in a study that included four different countries [4]. In Saudi Arabia, 50.6% of the participants of a study reported at least one adverse reaction from cosmetic products, including perfumes [5]. Fragrance substances added to cosmetic products and perfumes contributed to the majority of health adverse reactions caused by fragranced products [1,2].

Fragrance substances are natural or synthetic organic compounds with various attractive characteristics and effects. They are widely used in perfumes for their pleasant smell. A single perfume formula can contain several fragrance substances [2,6]. Many of the commonly used fragrances in perfuming are well-known contact allergens that have been attributed to different types of adverse reactions [2]. The EU Scientific Committee on Consumer Safety listed 54 fragrance substances and 28 natural extracts that have been identified as “established contact allergens in humans” [6]. Currently, there is no regulation in Saudi Arabia that requires the disclosure of perfume contents.

The growth rate of the perfume market in Saudi Arabia sharply increased by 25.9% in 2014, with total sales of about 3.5 Billion Saudi Riyals [7]. Taking into account the huge market volume, studies targeting the safety of perfumes in the Saudi market are still lacking. In addition, the prevalence of hypersensitivity reactions caused by perfumes among the public in Saudi Arabia have not yet been investigated. Therefore, the main objectives of this study were to identify the prevalence, nature, and associated risk factors of perfume hypersensitivity reactions among the general population in Saudi Arabia.

## 2. Materials and Methods

### 2.1. Study Design

A cross-sectional study was conducted among adults aged 18 years or older in October 2020. Residents of the capital cities of the five main regions in Saudi Arabia—the Central, Northern, Southern, Eastern, and Western Region—were included. This study has been reviewed and approved by the Human Research Ethics Committee of the Saudi Food and Drug Authority (Approval number: 2020_007).

### 2.2. Sample Size

We used the proportional quota sampling technique to ensure that respondents were demographically representative of the general population. The sample population was divided according to region and gender distribution of the general population, based on the latest published census data (2017) [8]. The sample size was calculated at a confidence level of 95%, and a 3% margin error; we estimated that a minimum sample size of 1068 participants would be sufficient. Moreover, we considered a 10% response rate from the total recruited sample of 10,680.

### 2.3. Recruitment Method

A list of participants generated from a governmental database (Saudi Food and Drug Authority) was chosen randomly based on region and gender, and the selected individuals were invited to participate in the study. Participants were invited using a text message (SMS) containing a short description of the study and the survey link. Each user had a unique device identifier linked to the research database; hence, the user could not submit information more than once. A consent letter in the first section of the survey was provided to all participants before their participation in the study survey. Data were obtained electronically, and no user could submit responses that were missing vital information. Phone interviews using CATI (computer assessed telephone interviewing) were conducted to follow up those who did not respond to the text message invitation.

### 2.4. Research Tool

We developed a survey comprised of four major parts. The first part aimed to assess the socio-demographic characteristics of the respondents, including age, gender, education level, monthly income, past medical history, previous dermatological disease, and the use of dermatological agents. The questions in the second part were about the participants’ perfume-buying behaviors, and it included the following: perfume-buying sources, used perfume types and brands, and the tendency to check perfume authenticity before buying. Perfume types were classified into four groups: Parfum, 20–30%, Eau de Toilette, 5–15%, Eau de cologne, 2–4%, and Perfume Oils [2].

In the third part, we assessed the history of perfume sensitivity by asking the participant if he/she had experienced any health problems when exposed to perfume products. If the answer was “Yes”, they were asked detailed questions about the type of reactions, duration, recurrence, need for medical care, and symptoms resolution. The fourth part included questions about the perceived causes of sensitivity and the reporting behaviors of those who developed sensitivity reactions caused by perfumes.

### 2.5. Outcome Measures

The primary outcome measures for the study were the prevalence and the associated risk factors of self-reported perfume sensitivity among adults in Saudi Arabia. Perfume sensitivity was identified by the report of any of the following symptoms: skin symptoms (redness, itching, blistering, discoloration), respiratory symptoms (difficulty in breathing, shortness of breath, cough), nasal symptoms (nasal congestion, sneezing, runny nose), or neurological (headache, dizziness, migraine, loss of coordination) [4,9].

Mild reactions were identified by the report of any of the listed symptoms without the need for medical care, whereas moderate/severe reactions were identified by the report of one or more of the skin or respiratory tract reactions, the need for medical care, and the duration of the reported symptom. Assessed associated sensitivity risk factors included participants’ demographics, past medical and dermatological histories, medication use, and used perfume types and brands. Secondary outcomes included perfume-buying behaviors, the perceived causes of sensitivity, and reporting behaviors among study participants.

### 2.6. Statistical Analyses

A descriptive analysis summarized by mean and standard deviation for continuous variables and percentages for categorical variables was conducted for all study variables. A binomial logistic regression analysis was used to identify perfume sensitivity risk factors, including demographics, past medical history, previous dermatological diseases, and the use of dermatological agents. Regression coefficients, odds ratios (ORs), 95% confidence intervals (CIs), and *p* values to quantify the associations between variables and study outcomes were reported. The statistical significance level was set at *p* < 0.05 (two-sided). All analyses were performed using R software (version 4.0.3).

## 3. Results

### 3.1. Social-Demographic Characteristics and Perfume Sensitivity Prevalence

A total of 1078 participants (mean age 36.7 years (SD ± 10.36)) completed the survey, with a response rate of 10%. From among the participants, 157 (14.4%) self-reported the experience of at least one perfume hypersensitivity reaction. More details of the participants’ characteristics are presented in Table 1.

### 3.2. Characteristics of Self-Reported Perfume Sensitivity Reactions

Among all participants with perfume sensitivity, respiratory symptoms and skin symptoms were the most reported reactions, with total rates of 40.1% and 35.7%, respectively. About 29% of participants reported nasal symptoms, whereas headache was the least reported symptom, with a rate of 10.4%. The most reported perfume type that caused sensitivity was Parfum (47.8%), and most of the reported products were unknown/counterfeit products (43.3%). Around 22% of participants with perfume sensitivity reported that their symptoms lasted more than a day, and 20% of them reported the need for medical care during their experience.

Mild reactions were reported in 82.2% of the participants, whereas moderate/severe reactions were found in 17.8% of the participants. Parfum and unknown/counterfeit products were the most reported type and brand of moderate/severe reactions, with percentages of 53.6% and 46.4%, respectively. Most participants (93%) reported a total resolution of the symptoms after stopping the perfume. Sensitivity recurrence was reported in 66.2% of participants, with 15.9% reporting the same experience with different perfume types and brands (Table 2).

### 3.3. Perfume-Buying Behavior

**Figure 1:**
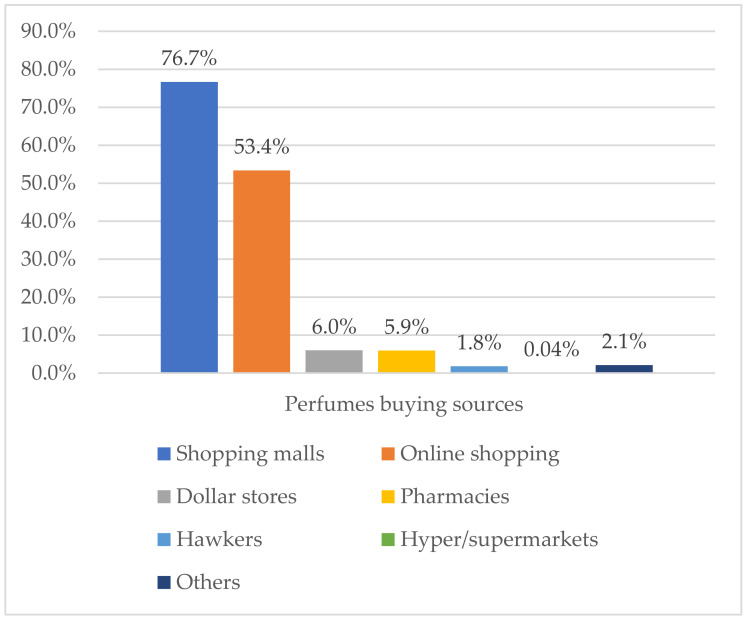
Perfume-buying sources among study participants (*n* = 1078).

Shopping malls and online shopping were the most reported sources of perfume buying among all study participants, with percentages of 76.7% and 53.4%, respectively (Figure 1). The most used perfume types were Parfum 66.4%, followed by Perfume oils 45.3%, Eau de Toilette 43.4%, and Eau de cologne 22.6%. Imported and domestic brand products were highly used among study participants, whereas the use of unknown/counterfeit products was reported in 15% of participants. About 45% of participants reported that they do not always verify perfume authenticity before buying. Full details are reported in Table 3.

### 3.4. Perfume Sensitivity—Associated Risk Factors

Multivariate logistic regression was used to identify the associated risk factors of perfume sensitivity. The model assessed the association of the demographic characteristics and past medical history with the risk of perfume sensitivity. It showed that a history of asthma diagnosis (OR = 3.2, 95%CI 1.88–4.37, *p* < 0.001) and the use of unknown/counterfeit perfume products (OR = 1.9, 95%CI 1.23–2.94, *p* < 0.003) were significantly associated with a higher risk of self-reported perfume sensitivity (Table 4).

### 3.5. Perfume Sensitivity Perceptions and Reporting Behaviors

**Figure 2:**
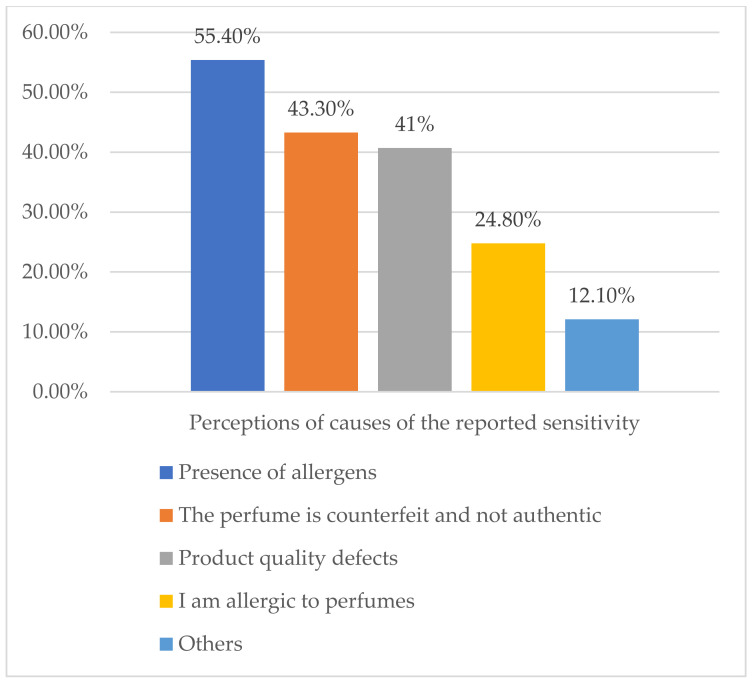
Perceptions of causes of the reported sensitivity (*n* = 157).

About 55% of those who self-reported perfume sensitivity attributed their experience to the presence of allergens in the perfume, whereas 24.8% thought that it was because they were allergic to perfumes (Figure 2). Only 1.3% reported their reactions to the responsible authorities. The main reason for the non-reporting was their lack of knowledge of the responsible authority. Other reasons are represented in Table 5.

## 4. Discussion

The results of this study indicate that 14.7% of the population in Saudi Arabia have experienced at least one of the perfume reactions. Our study investigated further and showed that previous history of asthma and the use of unknown/counterfeit products were significantly associated with the higher risk of perfume sensitivity among participants. Studies targeting the safety of perfume products exclusively are rare, and this study, to our knowledge, is the first epidemiological study that has targeted perfume products in the area.

A study conducted recently among 425 adults in the Eastern Province of Saudi Arabia reported that 50.6% of the participants had an adverse reaction from a fragranced product, including perfumes [5]. Internationally, a study conducted across four countries, including the United States, the United Kingdom, Australia, and Sweden showed that 32.2% of the participants reported sensitivity from fragranced products, including perfumes [4]. Furthermore, 34.7% of the population in the United States reported the experience of different health problems upon exposure to fragranced products [3]. In all these studies, perfumes were reported as one of the fragranced products that contributed to the reported health reactions [3,4,5]. The lower rate in our study can be justified by the fact that we targeted perfume products exclusively, while other studies included all types of fragranced products during their assessment.

Respiratory and skin symptoms were the most reported reactions among the participants in our study, with total rates of 40.1% and 35.7%, respectively. These results were consistent with the results of the international study, where they found respiratory and skin symptoms to be the most reported symptoms [3,4]. Reported skin symptoms in our study cannot be classified as allergic contact dermatitis without performing patch testing. However, positive patch test reactions to perfumes have been investigated previously [10,11]. A study conducted among female nurses showed that fifteen had a positive patch test reaction to a perfume mix of eight different perfume ingredients, and twelve of the fifteen reported previous perfume sensitivity [12]. Moreover, perfumes were the most frequent source of allergic contact dermatitis caused by fragranced products in a case-control study of eczema patients [13].

The significant associated risk factors identified in our study include a previous history of asthma and the use of unknown/counterfeit products. The association between asthma diagnosis and perfume sensitivity was previously identified [3,14]. A study that included 1137 asthmatics found that 64.3% of them reported one or more types of adverse health effects resulting from fragranced products [14]. In addition, the prevalence of fragrance sensitivity was higher among asthmatics than the general population in the United States. The association between the use of unknown/counterfeit perfume products and the risk of perfume sensitivity was not investigated before. Interestingly, our results showed that about 19% of those who reported perfume sensitivity in our study needed medical care for their reaction, which amplify the clinical irrelevance of the majority of the reported reactions. However, taking into consideration the nature of our study (self-reporting), the accuracy and the clinical significance of the reported reactions cannot be confirmed.

### 4.1. Strengths and Limitations

This is the first study in Saudi Arabia that assessed the adverse health reactions associated with exposure to perfumes. The participants in this study were recruited randomly from a governmental database and divided according to region and gender. The weighted distributions of respondents by age, gender, and region were consistent with 2018 estimates from the Saudi Arabian General Authority for Statistics of the five main regions in Saudi Arabia (Central region, Western, Eastern, Southern, and Northern regions) [8]. Despite the significant results, our study has some limitations. This was a cross-sectional study that was based on self-reported data from participants, risk of reporting bias exist. Due to the low response rate, two data collection methods were used (web-based survey and phone interviews); however, no significant difference was found between the results of these two methods. Lastly, we did not assess the history of previous food or drug allergies among participants.

### 4.2. Future Directions

The highly predicted expansion of the perfume market highlights the need for further research in order to assess the safety of the available perfume products in the market. Quantitative studies are recommended to determine the composition and the presence of allergenic fragranced substances in the available perfume products. Lastly, an assessment of the societal effects of the experience of perfume sensitivity can be considered in future studies to understand the social extent of the problem [15].

## 5. Conclusions

Our study reveals that a considerable number of the general population in Saudi Arabia reports having experienced adverse health reactions from perfume products. The results of our study offer an insight into the safety of current perfume products available in the Saudi market. Future quantitative studies that determine the composition and the presence of fragranced allergens in the available perfume products are highly needed.

## Figures and Tables

**Table 1:** Socio-demographic characteristics among study participants (*n* = 1078).

	Total Sample (%)	Self-Reported Perfume Sensitivity	
		Yes	No
**Overall**	1078 (100)	157 (14.6)	921 (84.4)
**Age**			
18–30	331 (30.7)	36 (22.9)	295 (32)
31–45	548 (50.8)	93 (59.2)	455 (49.4)
≥46	199 (18.5)	28 (17.9)	181 (18.6)
**Gender**			
Male	644 (59.7)	110 (70.1)	534 (58)
Female	434 (40.3)	47 (29.9)	387(42)
**Nationality**			
Saudi	1006 (93.3)	147 (93.6)	859 (93.3)
Not Saudi	72 (6.7)	10 (6.4)	62(6.7)
**Marital status**			
Single	340 (31.5)	50 (31.8)	290 (31.5)
Married	697 (64.7)	98 (62.4)	599 (65)
Divorced/widowed	41 (3.8)	9 (5.7)	32 (3.5)
**Education**			
High school or less	251 (23.3)	29 (18.4)	128 (13.9)
Bachelor/Diploma	624 (57.9)	102 (65.3)	640 (69.5)
Master/PhD	203 (18.8)	26 (16.3)	153 (16.6)
**Region**			
Central	292 (27)	58 (36.9)	234 (25.4)
West	335 (31)	45 (28.7)	290 (31.5)
Eastern Region	177 (16.1)	22 (14)	150 (16.3)
South	139 (12.9)	13 (8.3)	126 (13.7)
North	140 (13)	19 (12.1)	121 (13.1)
**Monthly income**			
Less than 5000 SR	153 (14.2)	27 (17.2)	126 (13.7)
5000–10,000 SR	227 (21.1)	28 (17.8)	199 (21.6)
10,000–15,000 SR	200 (18.6)	40 (25.5)	160 (17.4)
15,000 SR or more	197 (18.3)	31 (19.7)	166 (18)
I prefer not to say	301 (28)	31 (19.7)	270 (29.3)
**Previous dermatological diagnosis**			
No	875 (81.2)	108 (68.8)	767 (83.3)
Yes	203 (18.8)	49 (31.2)	154 (16.7)
Reported dermatological diseases *			
Eczema	82 (7.6)	17 (10.8)	65 (7.1)
Vitiligo	9 (0.83)	2 (1.3)	7 (0.8)
Psoriasis	26 (2.4)	4 (2.5)	22 (2.4)
Allergies	91 (8.3)	28 (17.8)	62 (6.7)
Acne	41 (3.8)	7 (4.5)	13 (1.4)
**Regular use of dermatological agents**			
No	1014 (94.1)	142 (90.4)	872 (94.7)
Yes	64 (5.9)	15 (9.6)	49 (5.3)
**Previous medical diagnosis**			
No	624 (77.5)	110 (70.1)	725 (78.7)
Yes	243 (22.5)	47 (29.9)	196 (21.3)
**Reported medical conditions ^§^**			
Diabetes Mellitus	88 (8.2)	8 (5.1)	80 (8.7)
Hypertension	81 (7.5)	14 (8.9)	67 (7.3)
Asthma	86 (8)	28 (17.8)	62 (6.7)
Heart disease	29 (0.07)	8 (5.1)	21 (2.3)
Autoimmune	18 (0.2)	5 (3.2)	13 (1.4)

* Among those who reported previous dermatological diagnosis. ^§^ Among those who reported medical conditions.

**Table 2:** Self-reported perfume sensitivity among study participants (*n* = 157).

		Participants, *n* (%)
**Reported perfume type**		
	Parfum 20–30%	75 (47.8)
	Perfume oils	35 (22.2)
	Eau de Toilette 5–15%	29 (18.5)
	Eau de cologne 2–4%	18 (11.5)
**Reported perfume brand**		
	Unknown/counterfeit-origin products	68 (43.3)
	Imported brands	37 (23.6)
	Domestic brands	49 (31.2)
**Reported reactions**		
	Respiratory symptoms	63 (40.1)
	Skin symptoms	56 (35.7)
	Nasal symptoms	45 (28.7)
	Neurological symptoms	17 (10.4)
	Others	9 (5.7)
**Reported sensitivity duration**		
	Less than an hour	53 (33.8)
	An hour to 24 h	69 (44)
	A day or more	35 (22.2)
**The need for medical care**		
	Yes	31 (19.7)
	No	126 (80.3)
**Sensitivity severity**		
	Mild	129 (82.2)
	Moderate/severe	28 (17.8)
**Sensitivity resolution**		
	Yes	146 (93)
	No	11 (7)
**Sensitivity recurrence**		
	One time only	53 (33.8)
	More than once with the same product	49 (31.2)
	More than once with different products from the same perfume type	30 (19.1)
	More than once with different products from different perfume types	25 (15.9)

**Table 3:** Perfume-buying behaviors among study participants (*n* = 1078).

		Participants, *n* (%)
**Used perfume types**		
	Parfum 20–30%	716 (66.4)
	Eau de Toilette 5–15%	468 (43.4)
	Eau de cologne 2–4%	244 (22.6)
	Perfume oils	488 (45.3)
**Used perfume brands**		
	Imported brands	837 (77.4)
	Domestic brands	738 (68.2)
	Unknown/counterfeit origins products	164 (15)
**Verifying perfume authenticity before buying**		
	Always	480 (44.5)
	Sometimes	288 (27.8)
	Never	310 (28.8)
**Why? (If the answer is “sometimes” or “never”**		
	I only buy from branded shops	313 (52.3)
	I do not care about this information	89 (14.9)
	I do not know where I can find this information	191 (31.9)
	I do not mind buying unknown/counterfeit perfumes	48 (8)
	Others	11 (1.8)

**Table 4:** Multivariate regression analysis of perfume sensitivity risk factors.

	OR	95% CI	*p* Value
Gender	1.8	0.87–3.79	0.115
History of dermatological disease	1.37	0.72–2.45	0.304
Regular use of dermatological drugs	1.16	0.54–2.38	0.685
History of skin allergy	0.88	0.94–2.81	0.091
History of asthma	3.2	1.88–4.37	<0.001
The use of domestic perfume products	0.9	0.59–1.37	0.626
The use of unknown/counterfeit products	1.9	1.23–2.94	0.003

**Table 5:** Perfume allergy perceptions and reporting behavior among participants (*n* = 157).

	Participants, *n* (%)
**Perception of causes of the reported sensitivity**	
Presence of allergens	87 (55.4)
Product quality defects	64 (40.7)
The perfume is counterfeit and not authentic	68 (43.3)
I am allergic to perfumes	39 (24.8)
Others	19 (12.1)
**Allergy incident reporting**	
Yes, to Saudi Food and Drug Authority	1 (0.6)
Yes, to Ministry of Commerce	1 (0.6)
No	155 (98.8)
Reasons for not reporting the incident *	
I do not know the responsible authority	99 (63.9)
I do not know how to contact the responsible authority	70 (45.2)
I do not care to report	25 (16.1)
Others	24 (15.5)

* Among those who did not report the incident.

## Data Availability

Not applicable.
